# Case of Partial Fracture of the Os Femoris

**Published:** 1826-07

**Authors:** 


### CASE OF PARTIAL FRACTURE.*

March 13th, 1826. J. Douglas, ætat. 2 years and six weeks, of a ricketty, unhealthy habit, was ill about a year ago, and treated for marasmus, after the plan of Dr. Hamilton, sen. with good effect.

About 2 months ago, the right thigh became much swollen, and the motions of the hip and knee-joint affected; near the middle of the femur an obscure sense of flexibility was perceived, not having altogether the character of fracture. With fomentations, stimulating embrocations, and, occasionally, cathartics, the tumour subsided to a certain extent, but at the end of three weeks the child died suddenly.

*Dissection.* The four great cavities tolerably sound—mesenteric glands somewhat enlarged—mucous coat of the intestines natural.

The right femur was very carefully dissected out—it had the anterior ricketty curvature, and near the middle of the fore part of the bone, was a small carious spot, about the size of a split-pea; the periosteum was elsewhere entire. Upon making a longitudinal section of the bone, a fissure was found extending from the carious spot, obliquely downwards, through the bone, for more than two-thirds of its diameter. The bone contained very little earthy matter (indeed this was the case with almost all the bones of the extremities,) its internal periosteum was very vascular, and the cells were filled with what looked much like coagulable lymph. The circular and longitudinal lamellæ, so peculiar to ricketty bones, were found very distinct here.

Mr. B. Bell makes some observations, in which he imagines this to be a spontaneous fracture, arising, not from any accident; as the patient had been for some time before the affection of the thigh appeared, confined to bed, but from "the carious spot," having exercised "some influence in modifying the constitution of the bone," and the local irritation therefrom having "had the effect of inducing irregular, and, perhaps, spastic contractions of the flexor muscles, so violent as to occasion partial fracture."

We are rather inclined to doubt whether this was a case of fracture, and for these reasons: The bones were, as Mr. B. observes, nearly all flexible—there was no apparent accident; nor do we think that spasm of the muscles would break the bone, inflamed as it evidently was, without producing such pain as must have made it known—in fine, the substance found in the cancelli, "resembling organizable lymph," renders it more probable that the fissure was the result of scrophulous action, inflammation, and absorption.

Does not scrophulous action render the vertebra perforated and spongy?

### RUPTURE OF ANEURISMS IN THE BRAIN.—M. SERRES.

M. Serres has divided apoplexies, accompanied by extravasation, into

* Edinburgh Journal of Medical Science, No. 11. April, 1826. Mr. Benjamin Bell.

